# Laceration of the Perineum, with Destruction of the Recto-Vaginal Septum

**Published:** 1862-02

**Authors:** C. C. F. Gay

**Affiliations:** Buffalo, N. Y.


ART. IV.—*Laceration of the Perineum, with destruction of the Recto-Vaginal Septum. Operation and Result, by* C. C. F. GAY, M. D., *Buffalo, N. Y.*

Mrs. P——, aged 31 years, of infirm health, had her perineum lacerated at the time of her first confinement, eight years since; again ruptured in a subsequent labor five years since, and again two and a half years since, the laceration extending through the sphincter and recto-vaginal septum.

The head of the last child was unusually large. Since her last confinement there has been an inability to control the contents of the bowels, and consequently frequent copious fluid dejections which have imposed upon her a necessity for medical treatment, which has been persevered in, for many months, with only temporary relief.

*Nov.* 4th, Mrs. P. returned from the country, having been under the medical treatment of Dr. R. Williams of Byron, Genesee County, N. Y., a most able and intelligent physician, who had attended her in one of her labors. She now returned to the city for the purpose of having the operation performed for radical cure. Catamenia appeared to-day.

15th.—Preparatory to the operation ordered Ol Ricini ℥i., which moved the bowels twice.

16th.—In the morning had three dejections, and took morphia gr. ½. Proceeded to perform the operation, assisted by Drs. White, Williams, Loomis, Eastman and Brown. Chloroform administered; placed the patient on the table in the position for lithotomy, I denuded the bowel portion, using sharp pointed scissors, to the extent of five-eights of an inch on either side the fissure, running up to a pointthree-fourths of an inch above its terminus, without wounding the mucus membrane of the bowel. Then with the knife denuded a sufficient amount in length of the labium, on either side, to the depth of nearly an inch, freely removing an abundance of tissue; afterwards dividing the sphincter by two incisions. This done, the surfaces of the bowel portion were approximated by two silver sutures, small wire, twisted loosely, leaving them an inch and a half long, turned up in the axis of the vagina.

The surfaces of the perineal portion were next brought together by three silver sutures, large wire, twisted loosely, and left several inches long. On passing one finger into the rectum and another into the vagina, the septum was found to be completely closed,

The operation lasted one hour and a quarter. Patient was now placed in bed, water dressings applied, and morphia gr. i given. Ordered opium grs. ij, every four hours, with nourishment of a concentrated character. In five hours the gum-elastic catheter was used and retained in situ.

17th.—Did not sleep much: labia dry and hot, somewhat painful; vomited once; pulse 80 per minute; continue opium every four hours, with quinine sulph. gr. ij. ter die.

18th.—Obtained some rest; pulse 80 per minute; bowels quiet, considerable flatus; bladder irritable; urine has been drawn off every four or five hours.

19th.—Comfortable; catheter removed; some purulent discharge per vaginum; inject tepid water; at 12½ o'clock had a convulsion; another at 3¼ P. M., lasting for a moment; had three or four prior to the operation; continue treatment, and also the use of the catheter.

20th.—Examined the perineum by placing the patient upon her side; wound looks well, as if uniting.

21st.—Discharge of blood from vagina, supposed to be the menstrual flow.

23d.—Considerable pain in perineum; wound looking well; discharge from vagina ceased; removed the middle perineal suture.

24th.—Introduced rectal tube and kept it in situ for the purpose of allowing the escape of flatus, and to prevent it passing through the edges of the wound which would tend to prevent union, if allowed.

25th.—Removed the remaining two perineal sutures; bladder very irritable and painful; remove the catheter whenever used.

26th.—Had a copious fluid dejection from the bowels, which passed out through the rectum without doing any injury to the parts.

27th.—Had another dejection from bowels; introduced finger into the vagina and find union has taken place; the anterior portion of perineum seems firm and united; rectal tube daily removed, cleaned and replaced.

*Dec.* 3d.—Wound is healing kindly; bowels have been quiet.

4th.—Union complete, except a small fistulous opening half an inch from the anus, about the size of a small goose quill; apply argt. nit.

5th.—Took this morning an injection of warm water.

6th.—Had two evacuations of bowels yesterday.

7th.—Removed one of the internal sutures, using for this purpose the vaginal speculum.

12th.—Sitting up; fistulous opening nearly closed.

16th.—Removed the remaining suture; discontine opium, and take morphia gr. ½, night and morning.

28th.—Rode out to-day; has complete control over the sphincter, and has a strong perineum of normal breadth and thickness.

It may be observed that in this operation, the quill sutures, recommended by Dr. Baker Brown, were not used; indeed they appear to me of no value whatever.

The operation was performed in the manner recommended by Dr. Marrian Sims, with the exception that Dr. S. does not consider it at all essential to the success of the operation to divide the sphincter; and I believe also, he immediately makes use of the rectal tube. The success of this case seemed to me mainly to depend upon the two sutures, which held together the bowel portion.

---

*A Loadstone which belonged to Benjamin Franklin.*—At a sale in London of the effects of the late Mr. Quackett, the microscopist, was a loadstone which belonged to Franklin. It was incased in brass, on which was the inscription, "Benjamin Franklin, Boston, N. E., 1779."

